# Machine learning models for predicting the risk factor of carotid plaque in cardiovascular disease

**DOI:** 10.3389/fcvm.2023.1178782

**Published:** 2023-09-22

**Authors:** Chengling Bin, Qin Li, Jing Tang, Chaorong Dai, Ting Jiang, Xiufang Xie, Min Qiu, Lumiao Chen, Shaorong Yang

**Affiliations:** ^1^ Health Management Section, The First People’s Hospital of Neijiang, Neijiang, China; ^2^ Department of Respiratory and Critical Care Medicine, The First People’s Hospital of Neijiang, Neijiang, China; ^3^ Special Inspection Department, The First People’s Hospital of Neijiang, Neijiang, China; ^4^ Laboratory Department, The First People’s Hospital of Neijiang, Neijiang, China

**Keywords:** machine learning, prediction model, carotid plaque, shap, cardiovascular disease

## Abstract

**Introduction:**

Cardiovascular disease (CVD) is a group of diseases involving the heart or blood vessels and represents a leading cause of death and disability worldwide. Carotid plaque is an important risk factor for CVD that can reflect the severity of atherosclerosis. Accordingly, developing a prediction model for carotid plaque formation is essential to assist in the early prevention and management of CVD.

**Methods:**

In this study, eight machine learning algorithms were established, and their performance in predicting carotid plaque risk was compared. Physical examination data were collected from 4,659 patients and used for model training and validation. The eight predictive models based on machine learning algorithms were optimized using the above dataset and 10-fold cross-validation. The Shapley Additive Explanations (SHAP) tool was used to compute and visualize feature importance. Then, the performance of the models was evaluated according to the area under the receiver operating characteristic curve (AUC), feature importance, accuracy and specificity.

**Results:**

The experimental results indicated that the XGBoost algorithm outperformed the other machine learning algorithms, with an AUC, accuracy and specificity of 0.808, 0.749 and 0.762, respectively. Moreover, age, smoke, alcohol drink and BMI were the top four predictors of carotid plaque formation. It is feasible to predict carotid plaque risk using machine learning algorithms.

**Conclusions:**

This study indicates that our models can be applied to routine chronic disease management procedures to enable more preemptive, broad-based screening for carotid plaque and improve the prognosis of CVD patients.

## 1. Introduction

Cardiovascular disease (CVD) is generally divided into several types, such as coronary heart disease (CHD), cerebrovascular disease, heart failure, hypertension and so on (1). CVD is one of the leading causes of death worldwide, with 17.9 million deaths in 2016 and predicted to increase to approximately 23.6 million deaths by 2030 (2, 3). Due to the changes in lifestyle, aging population and urbanization, the prevalence of CVD in China has significantly increased (4). Cardiovascular disease afflicts 20% of the adult population in China, accounting for more than 40% of all deaths (5). Therefore, it is crucial to identify and diagnose CVD early to reduce the burden on families and society.

Hypertension and atherosclerosis are two well-recognized risk factors for CVD (6, 7). Current evidence suggests that a person with hypertension has a significant lifetime risk of cardiovascular disease by age 30 (8). According to previous studies, over half of all CVD cases in China are related to hypertension (4). In addition, atherosclerosis refers to the formation of carotid plaques in the inner lining of medium and large arteries. The risk of CVD is 1.3 to 2.8-fold higher among people with carotid plaques than those without, according to several large cohort studies (9, 10). Carotid plaque screening was recommended as part of the CVD risk assessments by the 2016 European Guidelines on CVD Prevention in Clinical Practice (11). Hence, carotid plaques have become an important measure of risk in many clinical studies.

In computer science, machine learning (ML) is a kind of artificial intelligence (AI) that harnesses data-driven techniques to recognize patterns and predict behavior (12). ML has been widely applied in multiple medical and health fields, such as cancer (13), diabetes (14), cardiovascular disease (CVD) (15) and mental health (16). ML can be used to predict the prevalence and treatment efficacy of diseases. Generally, the use of machine learning models can improve patient safety and care quality and reduce medical expenses (17). Interestingly, machine learning can predict hypertension based on clinical indicators. In a Swedish study, LR was used to establish a model to study heart rate, memory, metabolic characteristics and their relationship with hypertension (18). Using LR, Elizabeth Held et al. developed a hypertension risk model based on age, sex, smoking, and genetic data (19). Moreover, bioinformatics and machine learning identified immune cell infiltration and diagnostic biomarkers of carotid plaques (20, 21). The XGBoost algorithm has been reported to be the best predictive model for primary stroke prevention in a Chinese study (22). However, the predictive models for recognizing carotid plaques in CVD have rarely been documented in the literature, and it is essential to build a model to directly predict the risk of carotid plaques.

This study aimed to identify the most effective prediction model for the formation of carotid plaque among Chinese adults through eight machine learning methods: logistic regression (LR), support vector machine (SVM), random forest (RF), MLP Neural Network, XGBoost (Extreme Gradient Boosting), decision tree (DT), K-Nearest Neighbors (KNN), and Naive Bayes model (NBM), to screen high-risk populations, take preventive measures and reduce the prevalence of CVD.

## 2. Materials and methods

### 2.1. Study design and data resource

The data used for the machine learning model in this study came from the physical examination center of a third-class hospital in southwest China from September 2020 to October 2021. The inclusion criteria were (1) age > 18 years. (2) Patients without coronary heart disease, stroke, heart disease, cancer and other serious diseases. Physical examination data were collected from 4,659 patients, including 1,692 patients with carotid plaques and 2,967 normal patients. The diagnosis of carotid plaque was based on a doppler ultrasound examination, and personal information was kept strictly confidential.

**Figure 1:**
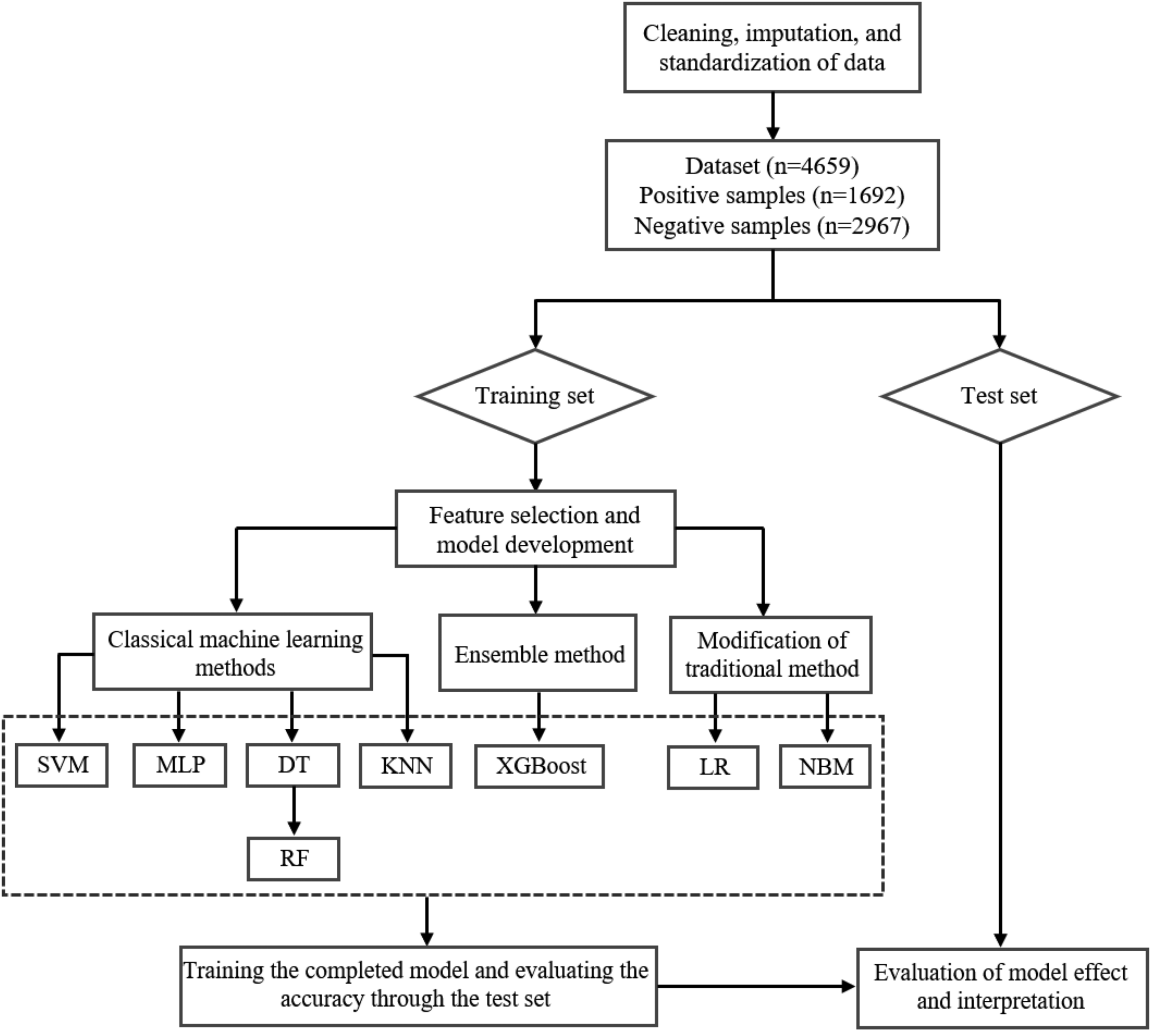
The flow diagram of data processing and model building process.

This study sought to build a risk prediction model for carotid plaque formation based on physical examination data and machine learning techniques to explore the risk factors of CVD and help clinicians in decision-making. The data processing and model-building process of machine learning is shown in Figure 1.

### 2.2. Feature selection

The constituent factors employed for the construction of the carotid plaque risk prediction model were acquired directly from outcomes of physical examinations, and demonstrated statistical significance in the course of data analysis (*P *< 0.05). According to the literature (23–25), we selected 14 variables related to carotid plaque formation. All direct variables were transformed into categorical variables to facilitate relevant calculations in machine learning models, and the specific details of the variables are presented in Table 1.

**Table 1:** Features description information.

Features	Type	Description
Age	Continuous	Age at health examination
Gender	Categorical	0: Female 1: Male
BMI	Continuous	BMI at health examination
SBP	Continuous	SBP at health examination
DBP	Continuous	DBP at health examination
Hypertension	Categorical	0: normal 1: hypertensive patients (SBP ≥ 140 or DBP ≥ 90 mmHg)
Family history	Categorical	0: no family history of hypertension 1: other situations
Smoke	Categorical	0: no long-term smoking habits 1: long-term smoking
Drink	Categorical	0: no long-term drinking habits 1: long-term drinking
Urea nitrogen	Categorical	0: normal (3.0–7.2 mmol/L) 1: up-regulation (>7.2 mmol/L)
Triglyceride	Categorical	0: normal (0.4–2.0 mmol/L) 1: up-regulation (>2.0 mmol/L)
Total cholesterol	Categorical	0: normal (3.1–5.7 mmol/L) 1: up-regulation (>5.7 mmol/L)
HDL-C	Categorical	0: normal (1.16–1.55 mmol/L) 1: up-regulation (>1.55 mmol/L)
LDL-C	Categorical	0: normal (1.8–3.36 mmol/L) 1: up-regulation (>3.36 mmol/L)

### 2.3. Machine learning algorithms

Eight predictive models were used to develop risk models based on the extracted features to predict the risk of carotid plaque formation.

Logistic regression (LR): LR is a generalized linear regression analysis model similar to linear regression modeling, and both are derived from the exponential distribution family. However, LR introduces a sigmoid function to normalize the dependent variable. Besides, the LR algorithm can directly probe the association between the categorical dependent variable and independent variables (26), commonly utilized in data mining, automatic disease diagnosis, economic prediction, etc.

Support vector machine (SVM): SVM is a generalized linear classification based on supervised learning that can create a hyperplane between two kinds of data to maximize the margin (27). SVM is a classifier with sparsity and robustness, which utilizes hinge loss to compute empirical risk and adds a normalized term in the solution system to optimize structural danger.

Random forest (RF): RF is a global learning technology based on decision tree analysis. The RF algorithm creates multiple decision trees using the data set obtained by bootstrapping the original data and randomly selecting a subset of variables in each step of the decision tree (28). Then, the model selects all predicted patterns of each decision tree. The RF classifiers were trained independently, contributing to the fast learning process.

The multilayer perceptron (MLP) Neural Network: MLP is also named Artificial Neural Network (ANN), a nonlinear mapping model. The MLP can have multiple hidden layers besides the input and output layers. The simplest MLP requires a hidden layer; the input, hidden and output layers can be called a simple neural network when they connect. A neural network consists of a series of functions derived from a bionic neural network that involve connecting multiple eigenvalues and combining them in a linear and nonlinear way (29).

XGBoost (Extreme Gradient Boosting): XGBoost is an optimized distributed gradient enhancement library that is efficient, flexible and portable. The algorithm for XGBoost is accomplished under the framework of Gradient Boosting (30). XGBoost also offers parallel tree promotion, which could precisely resolve numerous data science issues. For example, the same code runs in primarily distributed environments (Hadoop, SGE, MPI) and can solve issues beyond billions of examples.

Decision tree (DT): This decision analysis method is based on the occurrence probability of various situations; the probability (the expected value of the net present value is greater than or equal to zero) can be computed by generating a decision tree (31). The DT algorithm is a graphical method that objectively applies probability analysis. Its computational complexity is not high; the output results are easy to understand and insensitive to the loss of intermediate values.

K-Nearest Neighbors (KNN): KNN is a nonparametric statistical method for classification and regression. This method is simple and effective, of which the generated model is based on the entire training dataset. The prediction result of the new data point is obtained by searching the K instances (nearest neighbors) similar to the data point on the whole training set and summarizing the output variables of these K instances. The prediction effect of KNN is good and is not sensitive to outliers.

Naive Bayes model (NBM): NBM is a classification method based on the Bayesian theorem and independent assumption of feature conditions. The misjudgment rate is very low due to the knowledge of probability statistics used to classify the sample datasets (32). NBM comprises prior and posterior probabilities, which avoids the subjective bias of using only prior probability and the over-fitting phenomenon of using only sample information.

### 2.4. Model development

We randomly separated the data into a training set and validation set according to a ratio of 2:1; the validation set was further used to adjust the model parameters to establish several new models, while the training set was used to evaluate the performance of the eight predictive models. The main profiles of the training and validation sets are presented in Table 2. Linear and tree “weak” classifiers were used to adjust parameters and select the model with higher accuracy for output in the XGBoost algorithm. Considering that the total amount was relatively small, 10-fold cross-validation was used to reduce the impact of fitting on the model and adjust the parameters of machine learning models for obtaining optimal results when the model allows. All machine learning algorithms were operated in Python 3.8.

**Table 2:** The main profiles of the training set and test set.

Feature	Training set		Test set	
	Positive (*n* = 1,077)	Negative (*n* = 2,029)	Positive (*n* = 615)	Negative (*n* = 938)
Age (years)	66.9 (10.7)	51.5 (13.2)	66.63 (10.79)	51.81 (13.87)
Gender (male)	0.320 (0.466)	0.249 (0.432)	0.361 (0.480)	0.285 (0.451)
BMI	24.62 (3.13)	25.68 (2.95)	24.57 (3.40)	25.57 (2.75)
SBP	142.8 (20.2)	131.6 (18.1)	142.1 (20.6)	132.9 (17.7)
DBP	80.09 (11.4)	80.08 (11.4)	80.4 (11.9)	80.6 (11.1)
Hypertension	0.342 (0.338)	0.296 (0.312)	0.364 (0.301)	0.285 (0.316)
Family history	0.399 (0.489)	0.411 (0.492)	0.408 (0.491)	0.409 (0.491)
Smoke	0.940 (0.991)	0.849 (0.976)	0.945 (0.993)	0.846 (0.971)
Drink	0.851 (0.921)	0.887 (0.859)	0.813 (0.890)	0.882 (0.848)
Urea nitrogen	0.176 (0.381)	0.097 (0.296)	0.178 (0.382)	0.108 (0.311)
Triglyceride	0.225 (0.417)	0.339 (0.473)	0.255 (0.436)	0.311 (0.463)
Total cholesterol	0.166 (0.372)	0.146 (0.353)	0.160 (0.366)	0.139 (0.346)
HDL-C	0.304 (0.460)	0.196 (0.397)	0.271 (0.444)	0.201 (0.401)
LDL-C	0.329 (0.470)	0.324 (0.468)	0.322 (0.467)	0.315 (0.464)

### 2.5. Validation

Several evaluation criteria were adopted to validate the performance of the predictive models, including the area under the receiver operating characteristic curve (AUC), accuracy, sensitivity, specificity, precision, F-value and Youden index. Accuracy is the most commonly used classification performance indicator, indicating the model's accuracy. Sensitivity is the percentage of positive samples predicted to be positive. Specificity refers to the ratio of the number of negative samples identified by the model to the total number of negative samples. These parameters could be calculated as follows:

$$\mathrm{Accuracy}=\frac{\mathrm{TP}+\mathrm{TN}}{\mathrm{TP}+\mathrm{TN}+\mathrm{FP}+\mathrm{FN}}$$

$$\mathrm{Sensitivity}=\frac{\mathrm{TP}}{\mathrm{TP}+\mathrm{FN}}$$

$$\mathrm{Specificity}=\frac{\mathrm{TN}}{\mathrm{TN}+\mathrm{FP}}$$

$$\mathrm{Precision}=\frac{\mathrm{TP}}{\mathrm{TP}+\mathrm{FP}}$$

$$F-\mathrm{value}=\frac{2\times \mathrm{Precision}\times \mathrm{Sensitivity}}{\mathrm{Precision}+\mathrm{Sensitivity}}$$

Youdenindex=Sensitivity+Specificity−1

Where the TP is the number of positive samples determined as positive (true positive), FN is the amount of positive determined as negative (false negative), TN is the amount of negative determined as negative (true negative), FP is the number of negative samples judged as positive (false positive) (23). A receiver operating characteristic (ROC) curve was generated by obtaining the coordinate points under different thresholds and connecting various coordinate points with the true positive rate TP as the vertical axis and the false positive case FP rate as the horizontal axis. The AUC refers to the area under the ROC curve, which is less than 1. The larger the AUC value, the higher the accuracy of the classifier.

### 2.6. Model interpretation

Traditional interpretation methods for machine learning algorithms only reflect the importance of features and exhibit a limited ability to judge how the features are related to the final predicted result. To solve this problem, the Shapley Additive Explanations (SHAP) tool was used to better explain the prediction model, which is an additive interpretation model. The model generated a predicted value for each predicted sample, and a SHAP value was assigned to each feature in the sample. The absolute value reflects the importance of the feature, of which the positive and negative values reflect the positive and negative effects in predicting the risk of carotid plaque. A SHAP value greater than 0 indicated that the feature improved the predicted value and played a positive role.

## 3. Results

### 3.1. Baseline characteristics

Overall, the model error decreased as the number of features increased. However, in practical terms, it is challenging to implement an excessive number of features. Accordingly, the number of features should be adequate. Based on the risk factors of hypertension collected in a previous study (29), laboratory variables were added according to the results of physical examination, which is used to obtain more accurate predictive results. After feature extraction and selection, several variables such as age, gender, BMI, family history, smoke, alcohol drink, SBP, DBP, hypertension and so on were finally chosen as input features of the machine learning models. The above features were strongly associated with carotid plaque formation (*P *< 0.05). The results are summarized in Table 3.

**Table 3:** Baseline characteristics of the participants.

Features	Positive	Negative	*P*-value
N	1,692	2,967	/
Age	67.09 (10.89)	53.46 (13.44)	<0.001
Gender	563 (33.27%)	688 (23.19%)	<0.001
BMI	24.61 (3.22)	25.39 (3.05)	<0.001
SBP	142.7 (20.47)	133.54 (19.03)	<0.001
DBP	80.10 (11.57)	80.25 (11.38)	0.017
Hypertension	548 (32.38%)	869 (29.29%)	<0.001
Family history	662 (39.13%)	1,213 (40.87%)	0.005
Smoke	820 (48.47%)	1,308 (44.09%)	<0.001
Drink	835 (49.33%)	1,697 (57.19%)	<0.001
Urea nitrogen	309 (18.27%)	300 (10.11%)	<0.001
Triglyceride	397 (23.45%)	984 (33.16%)	<0.001
Total cholesterol	272 (16.08%)	421 (14.18%)	<0.001
HDL-C	494 (29.18%)	586 (19.75%)	0.039
LDL-C	552 (32.62%)	947 (31.92%)	<0.001

### 3.2. Model parameters

Based on the feature parameters selected above, we used the training set to determine the optimal hyperparameters of eight machine learning algorithms, including the value of k (the number of closest points) in the KNN method, tree depth in the DT model, learning rate in XGBoost algorithm, etc. The hyperparameter results of the various models with optimal performance are summarized in Table 4, including the default values in some machine learning algorithms.

**Table 4:** The concrete results of hyperparameter in each machine learning algorithm.

Machine learning algorithm	Hyper-parameter name	Value range	Value
Logistic regression	Penalty term	[None, L1, L2]	L2
SVM	Kernel	[rbf, linear, ploy]	linear
Random forest	n_estimator max_features bootstrap max_deep	[10,20,50,100,200] [3,4,5,6,7,8,9,10] [true,false] [5,6,7,8,9,10]	100 7 false 8
MLP	Solver Activation Hidden_layer_sizes	[SGD, Adam, RMSprop] [relu,logistic,tanh] –	Adam Relu =[10,100,10]
XGBoost	Booster n_estimators Learn rate	[gbtree, gblinear] [20,50,100,200] [0,1,0.01.0.001]	Gbtree 100 0.1
Decision tree	Criterion max_features max_deep	[gini, ID3, C4.5] [6,8,10,12,14] [6,8,10,12]	Gini 12 8
KNN	N_neighbors Algorithm metric	[5,15,30,50,100,200] [kd_tree, ball_tree] [Manhattan distance, Euclidean distance]	30 kdtree Euclidean distance
Naive Bayes	Model	[GaussianNB, MultinomialNB, BernoulliNB]	GaussianNB

### 3.3. Comparison of model performance

**Figure 2:**
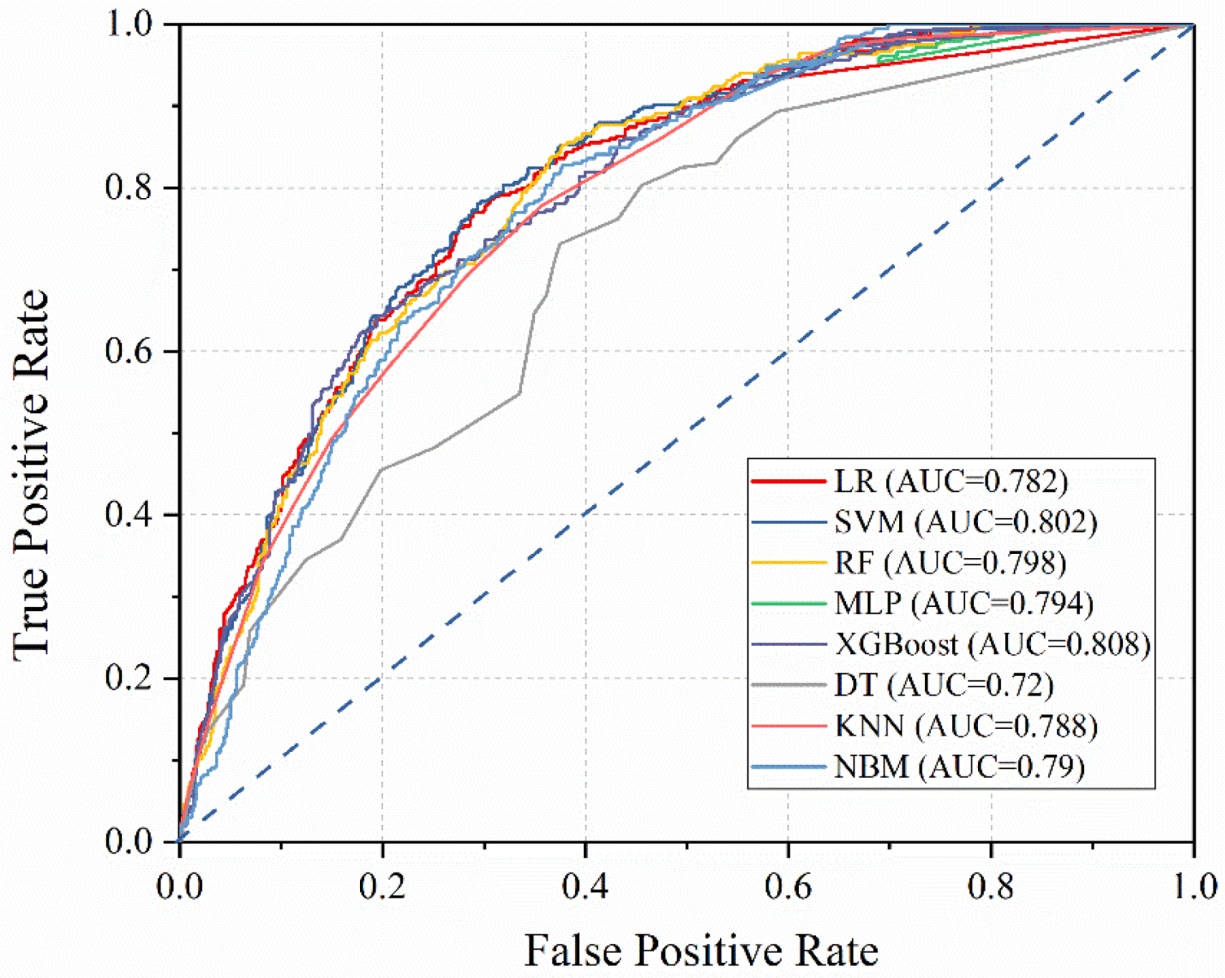
ROC curves of the eight machine learning models.

After building models based on the training set, their performance was assessed using the test set (Table 5). Interestingly, the integrated algorithm XGBoost outperformed all models, with an AUC value of 0.808. In contrast, the DT model was associated with the worst performance (AUC value 0.72). The ROC curves of the eight machine-learning models are shown in Figure 2. Besides, the confidence interval (CI) of LR, SVM, RF, MLP, XGBoost, DT, KNN and NBM is [0.768, 0.796], [0.790, 0.814], [0.781, 0.810], [0.775, 0.789], [0.796, 0.815], [0.704, 0.770], [0.738, 0.780], [0.747, 0.785], respectively. According to the results of CI, it is obvious that the results of our models are stable and reliable.

**Table 5:** The performance of each machine learning models.

Model	AUC	Accuracy	Sensitivity	Specificity	Precision	*F*-value	Yuden Index
LR	0.782	0.719	0.562	0.809	0.628	0.593	0.371
SVM	0.802	0.738	0.633	0.794	0.623	0.628	0.427
RF	0.798	0.746	0.632	0.802	0.615	0.623	0.434
MLP	0.794	0.728	0.605	0.793	0.610	0.608	0.398
XGBoost	0.808	0.749	0.724	0.762	0.632	0.675	0.486
DT	0.72	0.72	0.563	0.812	0.637	0.598	0.375
KNN	0.788	0.729	0.523	0.84	0.637	0.574	0.363
NBM	0.79	0.705	0.566	0.786	0.608	0.586	0.352

**Figure 3:**
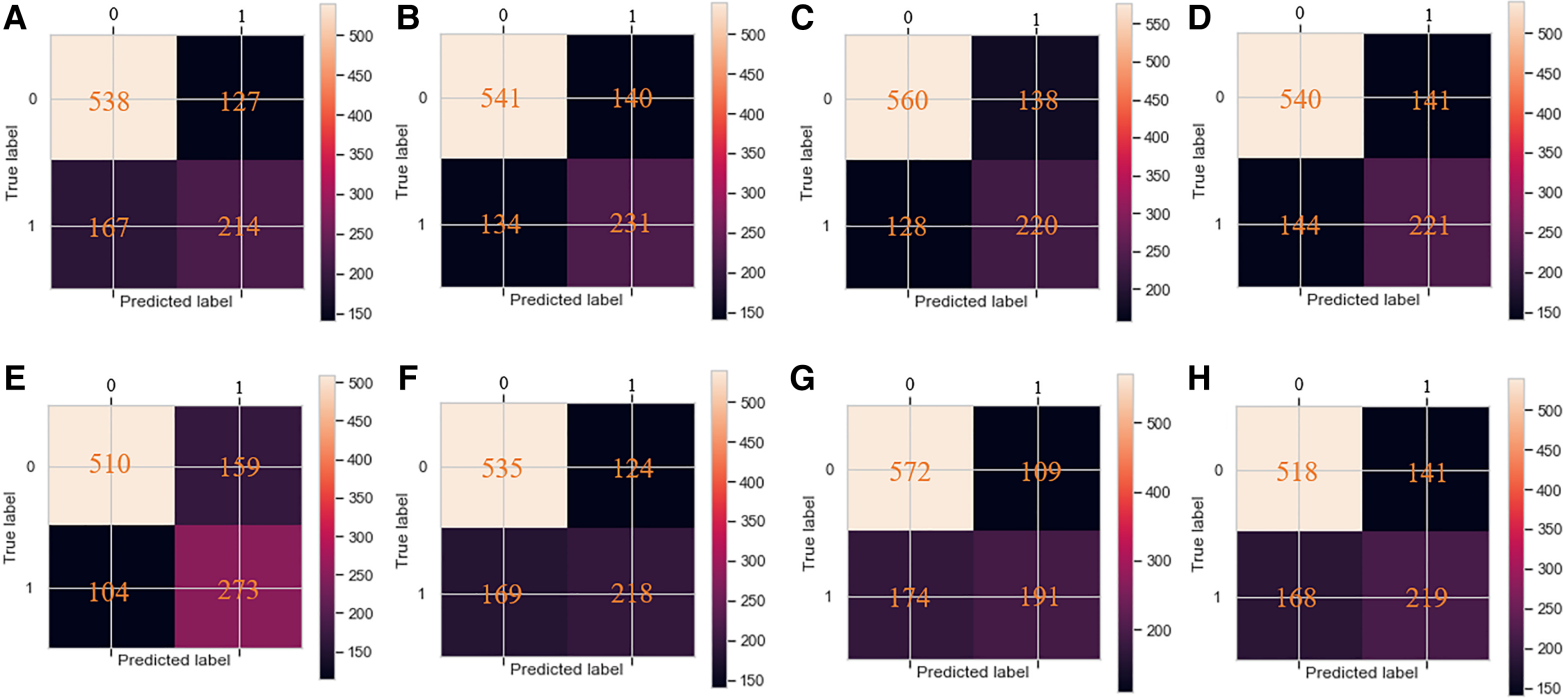
The confusion matrix of the eight machine learning models, including (**A**–**H**) LR, SVM, RF, MLP, XGBoost, DT, KNN and NBM, respectively.

The classification confusion matrix of the eight prediction models is shown in Figure 3. Among the top 4 models, the XGBoost model yielded the best performance in terms of TN, FP, FN and TP (510, 159, 104 and 273) compared to the SVM model (541, 140, 134 and 231), RF model (560, 138, 128 and 220) and MLP neural network model (540, 141, 144 and 221, respectively).

In addition to the AUC value, the accuracy, sensitivity and specificity of models are important indicators for evaluating a model's performance. For the four models with the best performance mentioned above (XGBoost, SVM, RF and MLP), the accuracy rate was 0.749, 0.738, 0.746 and 0.728, the sensitivity was 0.724, 0.633, 0.632 and 0.605, and the specificity was 0.762, 0.794, 0.802 and 0.793, respectively. Based on the above result, the XGBoost model yielded the best comprehensive performance.

**Figure 4:**
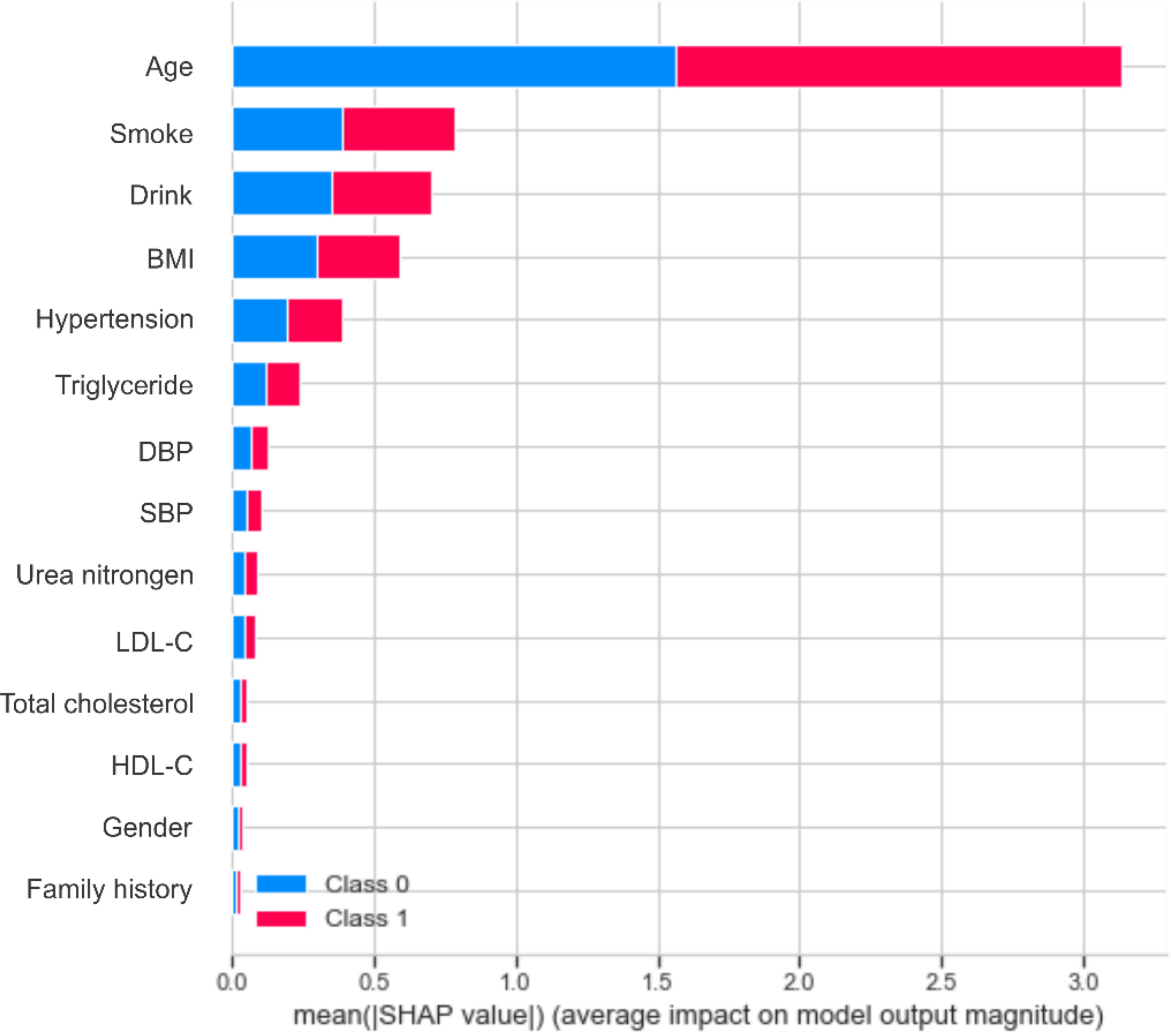
The feature importance ranking based on the SHAP value for the XGBoost model.

**Figure 5:**
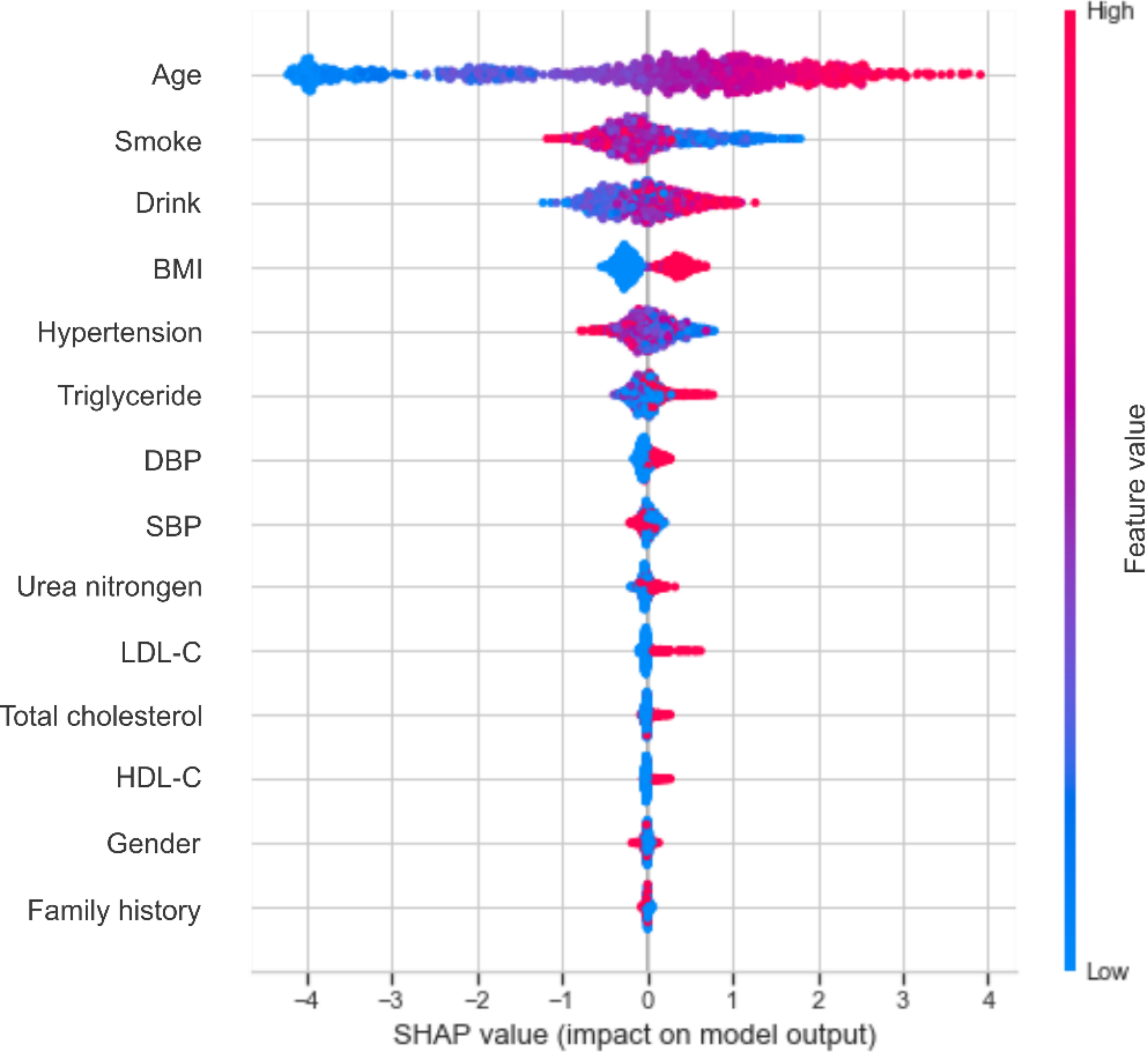
The SHAP summary chart of the important risk factors. Each dot represents a sample, with the red color implying a high feature value and blue one implying a low value. A higher SHAP value means a higher risk of incident carotid plaque.

Based on the performance of the XGBoost model, we computed the importance of each feature based on the absolute SHAP value (Figure 4). The negative and positive contributions of the feature are indicated in blue and red, respectively. The SHAP summary plot is also presented in Figure 5. The most important characteristic was age, followed by smoke, alcohol drink, BMI, hypertension and triglyceride, SBP, DBP, urea nitrogen, LDL-C, total cholesterol, HDL-C, gender and family history.

## 4. Discussion

Cardiovascular diseases are a group of disorders involving the heart or blood vessels and remain a major cause of death and disability worldwide, accounting for about one-third of deaths each year. Hypertension is a primary risk factor for cardiovascular disease, and the risk assessment of hypertensive patients is the basis for developing primary and secondary prevention measures. Nowadays, evaluating target organ damage is an important part of the evaluation of hypertensive patients. Embodying subclinical target organ damage in hypertensive patients, carotid plaque formation can reflect the severity of atherosclerosis in the body and is an important risk factor for major cardiovascular diseases such as myocardial infarction and stroke. Therefore, a direct risk assessment and prediction of carotid plaque formation would be more effective. To our knowledge, most risk prediction models associated with cardiovascular disease have been based on hypertension (33–35). Herein, we established a carotid plaque risk prediction model using machine learning to lay the groundwork for hypertension and cardiovascular disease prevention in China, given the scarcity of related studies.

In the past, the early identification and treatment of diseases were highly challenging, often requiring the consensus of many human medical experts. With the advent of artificial intelligence, building disease risk prediction models based on machine learning has become a new method of early identification of high-risk populations. From the point of view of saving medical costs, the predictive models could realize early detection and intervention, which could identify individuals at risk of developing carotid plaque before they exhibit symptoms or complications. Early detection allows for timely interventions, such as lifestyle modifications or medication, which can help prevent the progression of the disease and the need for more expensive treatments or hospitalizations in the future. Then, the predictive models could help identify individuals who are more likely to develop carotid plaque, allowing healthcare providers to prioritize resources and target screening efforts. By focusing on high-risk individuals, unnecessary screenings and tests for low-risk individuals can be minimized, thereby reducing overall healthcare costs. For example, according to the charging standards of the hospital (a tertiary A-level hospital at the prefecture-level city), the cost of physical examination in this project is only 72 RMB/person, while the charge for a carotid ultrasound is 185 RMB/person. Based on the optimal calculation, the 4,659 patients counted in this work could save more than 520,000 RMB, and this is only the conclusion obtained from the data of one hospital. Finally, the predictive models could analyze a wide range of patient data, including demographics, medical history and lifestyle factors to generate personalized treatment plans. The healthcare resources can be utilized more efficiently, potentially reducing costs associated with ineffective or unnecessary treatments.

In this study, eight machine learning algorithms were used to establish a risk prediction model for carotid plaque, and the input features were from physical examination data that are convenient to obtain. Among the eight models, XGBoost yielded the best performance (AUC value 0.808 and accuracy 0.749), suggesting that the machine learning models based on 13 key features were reliable and practical.

Compared with traditional statistical methods, machine learning models enable the most accurate predictions possible due to their ability to successfully build predictive models using small amounts of data with high feature dimensions but exhibit limitations, including poor interpretability and the inability to get a complete picture of the internal structure of the model, which is also known as the “black box”. This is further explained by the fact that as the complexity of the defined mathematical objects (neural networks) increases, we do not have a perfect theory to describe the expressibility, training dynamics, and various other properties of artificial neural networks at the whole system level. To improve the transparency of the model, we applied the SHAP method for the XGBoost algorithm to quantitatively explain the contribution of each feature to the whole model. This approach could measure the impact of each feature on the predictive model.

Age, smoke, drink and BMI were the top four most important characteristics, consistent with the literature (28, 29, 36, 37). Early identification of key risk factors is important for risk assessment and prevention of carotid plaque, hypertension and cardiovascular disease. In the present study, age accounted for the largest proportion of the feature weight. It has been shown that the elasticity of blood vessels deteriorates with age (38), which could prompt the development of atherosclerosis. An article published in The Lancet Global Health revealed that 200 million Chinese people are expected to suffer from carotid plaque by 2020 (39). The detection rate of carotid plaque in patients over 45 years of age with diagnosed stroke or transient ischemic attack is 80%, which is close to 100% at age 60. Smoke and drink ranked second and third in the whole features. An increasing body of evidence suggests that nicotine in cigarettes can increase the level of low-density lipoprotein that causes atherosclerosis, reduce the level of high-density lipoprotein that protects arteries from atherosclerosis, and increase blood pressure (40, 41), leading to atherosclerosis. Heavy drinking can decrease cerebral blood flow, damage the liver, and affect lipid metabolism (42). Besides, alcohol increases cardiac excitability, leading to increased heart rate and contraction, conducive to increased blood pressure.

Besides, BMI is an important risk factor. There is an increasing consensus that obesity can heighten peripheral blood vessel resistance, increasing blood pressure and further contributing to carotid plaque formation (43, 44). A study showed that the arteries of obese subjects began to stiffen when they were teenagers (45). Solving the problem of obesity as early as possible is critical for vascular health, and regular exercise to keep BMI in the normal range is necessary. Among the numerous indicators of obesity, BMI is most closely related to the formation of carotid plaque (46), followed by hypertension, triglyceride, DBP, SBP, urea nitrogen, LDL-C, total cholesterol, HDL-C, gender and family history.

The XGBoost machine learning model established in the present study exhibited good predictive performance with important public health implications. Data from routine physical exams can help doctors screen people at high risk for carotid plaque and take preventive measures to prevent them from developing more dangerous cardiovascular diseases. In addition, XGBoost and other models used in this study are nonlinear ensemble algorithms. There is no need to select variables in advance, even the number of potential variables is large (47). Potential variables can be further discovered through large-scale population data, which is also a big advantage brought by machine learning.

Despite the above promising findings, the current research still has some limitations. Firstly, the dataset utilized for model training in this work was obtained from the cross-sectional data of physical examinations, which cannot predict the absolute risk of disease and limits the generalizability of the results. Besides, the data from the cross-sectional study was collected at the same point in time, so it does not reflect causation or temporal correlation. Secondly, our data came from a third-class hospital in southwest China, suggesting that our conclusions only reflect the relevant characteristics of the residents in a specific area. Given that diet and climate vary greatly in different regions, it remains unclear whether the established machine learning models can be extended to the whole country. Thirdly, the sensitivity of the model in this work is not particularly high, which may result in some omissions when using it as a screening tool. We expect to improve the overall performance of the predictive models by using more training data and improving the machine model algorithm in the future. Moreover, the number of features was relatively small, and the prediction models may lack universality. Generally, a greater number of features lead to a more accurate model.

## 5. Conclusions

Overall, we developed and compared eight machine learning models for predicting carotid plaque risk. The results showed better performance of the XGBoost algorithm than other machine learning methods with an AUC, accuracy, sensitivity and specificity of 0.808, 0.749, 0.724 and 0.762, respectively. In addition, age, smoke, drink and BMI were the features with the most significant weight in the prediction model. The training set for all models consisted of routine physical examination results, which can be easily applied to chronic disease health management systems and could assist clinicians in targeted carotid plaque prevention and early intervention.

## Data Availability

The raw data supporting the conclusions of this article will be made available by the authors, without undue reservation.
